# Tensiometric evaluation of the effect of lowfrequency electric stimulation on healing Achilles tendons in rats^1^

**DOI:** 10.1590/ACB351103

**Published:** 2020-12-18

**Authors:** Sharbo Martins Casagrande, Maria de Lourdes Pessole Biondo-Simões, Lucas Freitas Berti, Rogério Ribeiro Robes, Rachel Biondo-Simões, Thaísa Sami Nakadomari, Lucélio Henning

**Affiliations:** I Fellow Master Degree, Postgraduate Program in Clinical Surgery, Universidade Federal do Paraná, Curitiba-PR, Brazil. Conception and design of the study; acquisition, analysis and interpretation of data; technical procedures; manuscript writing; critical revision; final approval.; II Full Professor, Department of Surgery, and Permanent Professor, Postgraduate Program in Clinical Surgery, Universidade Federal do Paraná, Curitiba-PR, Brazil. Conception and design of the study; acquisition, analysis and interpretation of data; technical procedures; critical revision; final approval.; III Associate Professor, Department of Mechanical Engineering, Universidade Tecnológica Federal do Paraná, Curitiba-PR, Brazil. Conception and design of the study; acquisition, analysis and interpretation of data; technical procedures.; IV Physician, Veterinary Hospital, and Fellow Master degree, Postgraduate Program in Clinical Surgery, Universidade Federal do Paraná, Curitiba-PR, Brazil. Technical procedures.; V PhD,Oncology Service, Hospital Angelina Caron, and Fellow Master Degree, Postgraduate Program in Clinical Surgery, Universidade Federal do Paraná, Curitiba-PR, Brazil. Acquisition of data, technical procedures.; VI Fellow Master Degree, Postgraduate Program in Clinical Surgery, UFPR, Curitiba-PR, Brazil. Technical procedures.; VII Graduate student, Universidade Federal do Paraná, Curitiba-PR, Brazil. Technical procedures.

**Keywords:** Electric Stimulation, Achilles Tendon, Wound Healing, Biomechanical Phenomena, Rats

## Abstract

**Purpose:**

To evaluate the effects of low-frequency electric stimulation on biomechanics following surgical treatment of the Achilles tendon in rats.

**Methods:**

Forty-two rats were divided into two groups. One was given electric stimulation and the other was not. All were submitted to Achilles tenotomy and tenorrhaphy performed with a modified Kessler stitch. The experiment group underwent electric stimulation with 2 Hz, a nonpolarized current of 1 mA intensity for 14 days. The animals were euthanized at 2, 4 and 6 weeks for the biomechanical study.

**Results:**

The work performed, that is, the tendon’s capacity to absorb energy until rupture, was greater in the electrically stimulated group in the 2nd (p = 0.032) and in the 6th week (p = 0.010). The maximum tension, which is the capacity to support a load, was higher in the treated group in the 2nd (p = 0.030) and the 6th week (p = 024). These results indicate greater resistance of the electrically stimulated tendons. An analysis of the elastic module showed no differences.

**Conclusion:**

Low-frequency electric stimulation increased the resistance of the tendons at 2 and 6 weeks of evolution in rats.

## Introduction

Achilles tendon lesions are among the most frequent tendon lesions and the most common in the lower limbs, accounting for 20 to 50% of all lesions. They have become more common in recent years, mainly as a result of traumas associated with practicing sports^1^–^3^.

Achilles lesions present high levels of morbidity and complications. As they occur in a hypovascular area, and thus with a low-quality healing process, there is a high rate of recurring ruptures and a high number of complications, in addition to prolonged rehabilitation^1^,^3^.

The treatment protocols undergo constant changes. Even with numerous random clinical studies, no consensus has been reached. However, many meta-analyses have shown that the recurrence rate after surgical treatment is significantly lower than after nonsurgicaltreatments^4^–^7^.

Surgical complications are generally related to the healing of the tendon or the skin and can result in new early or late ruptures, dehiscence, infections and even skin necrosis^1^,^2^,^4^,^5^,^7^.

Studies regarding electric stimulation in tissue healing have found different results. Many authors have shown good results with better cutaneous perfusion, increased local circulation and better healing in a wide range of tissues such as tendons, skin, bones and ligaments^8^,^9^. Nevertheless, Folha *et al.* 10 presented inconclusive results. The factors that hinder a comparison of the tests that have been made available include the diverse parameters used for electric stimulation^9^,^11^.

Ahmed *et al.* 12 studied Achilles tendons in rabbits and showed better healing in those submitted to low-frequency electric stimulation (10 Hz) and a polarized current (anode and cathode). Studies in animals have already shown that electric stimulation facilitates osteogenesis in fractures of the fibula in rabbits and the epithelialization of surgical wounds in pigs^13^,^14^.

Experimental studies in animals, both *in vivo* and *in vitro,* have shown superior results following electric stimulation, with better resistance to tensiometric tests. In the histopathological analysis, they found a less granulation tissue and more aligned collagen bundles^12^,^15^. An increase in the concentration of adenosine triphosphate (ATP), amino acid uptake and protein synthesis in human and animal skin fibroblasts has also been demonstrated^16^,^17^.

Clinical studies in humans have shown that the application of low-intensity microcurrents facilitates the healing of skin ulcers, reducing the amount of debridement, keeping them healed for longer, and also leads to a lower infection rate^18^,^19^. In neural regeneration, there appears to be a consensus concerning the benefits of low-frequency electric stimulation^20^–^23^. Meanwhile, high-frequency electric stimulation seems to have deleteriouseffects^24^.

To achieve better functional results in the resolution of tendon lesions, it is necessary to seek therapies that facilitate the healing process: less complex options at a low cost. As electric stimulation is a simple and easily accessed therapy, many people could benefit from the improved tissue healing process, with better results, fewer complications and faster rehabilitation.

The aim of this work was to evaluate the influence of low-frequency electric stimulation on the healing of Achilles tendons in rats following surgical repair through tensiometric study.

## Methods

### Ethical evaluation

The present study was conducted following analysis and approval by the Ethics Committee on the Use of Animals (CEUA/BIO – UFPR) at the Department of Biological Sciences of the Universidade Federal do Paraná, with Approval Certificate No. 1170. The study complied with the norms of Federal Law 11.797 of 08 October 2008, regulated by Decree 6.899 of 15 July 2009.

### Sample, accommodation conditions and constitution of the groups

Forty-two male Wistar rats (*Rattus norvegicus albinus,* Rodentia, Mammalia) from the UFPR animal facility (vivarium) were used in this study, aged 200 ± 5 days and weighing 517.4 ± 57.3 g.

The animals were kept in polypropylene boxes, suitable for the species, containing white shavings (changed daily), with four animals per cage. They were provided with water and standard commercial feed suitable for the species *ad libitum*. The light-dark cycle was 12 h, the room temperature was 20 ± 2 °C and the relative humidity was the same as that of the environment, i.e., with no artificialregulation.

The animals were randomly allocated to two groups. Group A was the control group (tenotomy and suture)and Group B was the experiment group (tenotomy, suture and electric stimulation at 2 Hz). Both groups were divided into three subgroups, with 7 rats in each subgroup, for evaluation after 2, 4 and 6 weeks.

### Surgical procedure

The surgical procedure was conducted at the Laboratory of the Discipline of Surgical Techniques and Experimental Surgery of the Federal University of Paraná, and the postoperative maintenance in a connecting room.

The anesthesia and analgesia were handled by a veterinary doctor. Anesthesia was initiated with preanesthetic medication via an intramuscular injection of ketamine hydrochloride 50 mg/kg combined with xylazine hydrochloride 2 mg/kg. The anesthetic medication was induced through inhalation with 1% isoflurane and maintenance with the same drug at 1.5% under a mask with 100% oxygen^25^.

**Figure 1:**
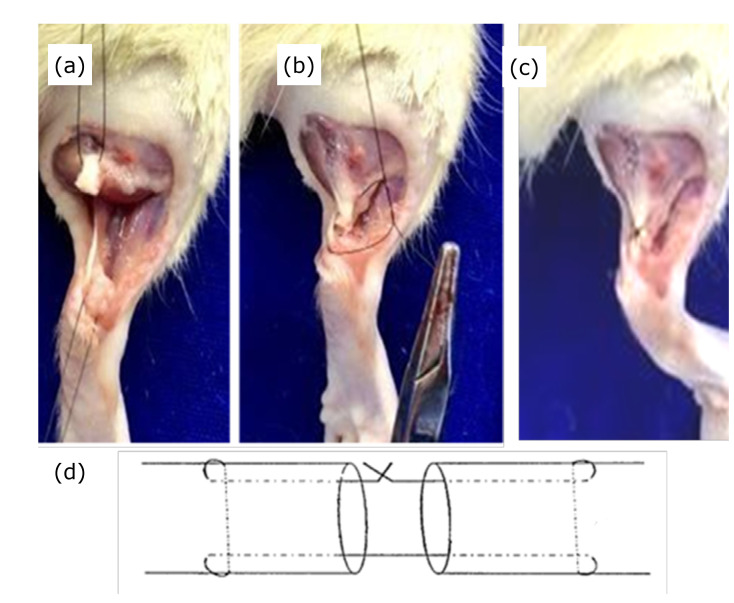
Surgical procedure. (a) Achilles tendon isolation and complete tenotomy, followed by (b and d) tenorrhaphy with modified Kessler stitch, and (c) completed tenorrhaphy.

The animals remained unfed for 4 h prior to the surgical procedure, with no restrictions on water. They were weighed and the posterior region of their foot was shaved using an electric device. After the antisepsis procedure, with polyvinylpyrrolidone-iodine, the skin was incised, the Achilles tendon was isolated and the complete Achilles tenotomy (lateral and medial band) was performed. Tenorrhaphy was then performed with 6-0 nylon monofilament thread using modified Kessler stitches and skin synthesis with simple stitches with 4-0 nylon monofilament thread(Fig. 1).

Group A was submitted only to tenotomy followed by the repair. In Group B, immediately after the surgical procedure, the first session of electric stimulation took place.

The postoperative analgesia, following recovery from the anesthetic procedure, was in the form of intramuscular administration of sodium dipyrone monohydrate 50 mg/kg and was maintained every 12 h^25^. The animals were returned to their cages, where they remained, and were given feed and water *ad libitum.*

### Electric stimulation

The electric therapy session was analgesic^26^,^27^. The animals in Group B animals were subjected to anesthetic induction on a daily basis via inhalation with isoflurane 1 to 1.5% under a mask associated with 100% oxygen and had a session of electric stimulation within the parameters established for the study lasting 20 min for 14 days.

**Figure 2:**
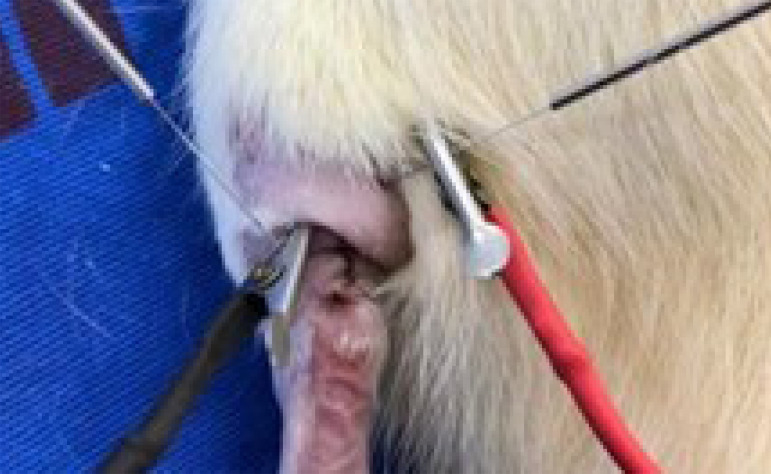
Needle insertion (0.25 × 30 mm) proximal and distal to tenorrhaphy of the Achilles tendon for electric stimulation.

The device used for electric stimulation was the Nkl 608 digital, serial number EM 4544, approved by the Brazilian National Sanitary Surveillance Agency (Anvisa), reviewed and calibrated by the manufacturer. An alternating current was used, and thus, without the formation of a positive and negative pole, a nonpolarized current, biphasic rectangular pulse of 600 µs, intensity of 1 mA, frequency of 2 Hz, and acupuncture needles 0.25 × 30 mm, distal and proximal to the surgical site. Peak output voltage adjusted automatically (Fig. 2).

### Euthanasia

The animals in the subgroups were euthanized after 2, 4 and 6 weeks. The tendons were completely resected, on the left side, for the tensiometric study.

Euthanasia was performed under anesthesia, in accordance with the protocol described in the CONCEA *Euthanasia Practice Guidelines* (2013) and the *Brazilian Guide to Good Euthanasia Practices in Animals of the Federal Council of Veterinary Medicine* (2013). Euthanasia was induced with inhaled anesthetic after venipuncture of the caudal vein and administration of sodium thiopental, 15 mg IV and 10% potassium chloride IV.

### Tensiometric study

The biomechanical study was conducted at the Graduate Department of Universidade Tecnológica Federal do Paraná (UTFPR) with a universal testing machine for the study of traction EMIC DL-10000 and analysis using Tesk EMIC software. A 50-N load cell was used with claws for polymeric materials to reduce the clamping force of the claws on the samples. The properties analyzed were the work performed for the rupture, which is obtained by the area under the specific stress versus strain curve, the maximum stress, the maximum load and the elastic modulus, which is obtained by the slope of the curve in the linear part of the test. To determine the maximum stress, the cross-sectional area was taken into account. The parts were photographed on graph paper from the front and in profile and Image-Pro Plus Version 4.5 software was used to determine the dimensions of the tendons in two directions (profile and front) of the tendon diameter. The model for the area of an ellipse was used to calculate the area of the tendon. The tendons were fixed in the test claws, using a 0.4-mm thick polymeric wrapper and cyanoacrylate super glue to enable fixation in the claws without causing damage to the tissue. For the beginning of the test, a preload (2 N) was used to adjust and accommodate the sample in the claw so that the test could then be conducted at a speed of 5 mm/min.

The values of the work that was done, measured in megajoules per cubic meter (MJ/m^3^), show the tendon’s capacity to absorb energy until rupture. The maximum stress values, measured in megapascals (MPa), show the tendon’s capacity to support loads, and use the tendon thickness in the calculation and the maximum load values. The results with the highest values indicate a more resistant fabric. The maximum load values, measured in Newtons (N), are correlated with the maximum stress. However, they do not take into account the tendon area, and therefore make the maximum stress value preferable for comparisons between samples. The elastic modulus values, measured in megapascals (MPa), evaluate the elasticity/rigidity of the tissues.

### Statistical analysis

The results of the quantitative variables were described by mean, standard deviation, median, minimum values and maximum values. Categorical variables were described by frequency and percentage. For the comparison of the three groups evaluated at 2, 4 and 6 weeks, with regard to quantitative variables, the Kruskal–Wallis nonparametric test was used. At each evaluation, the control and experiment groups were compared using the Mann–Whitney nonparametric test. Fisher’s exact test was used for the comparative analysis of categorical variables. Values of p < 0.05 indicated statistical significance. The data were analyzed using the computational program Stata/SE v.14.1 (StataCorpLP, USA).

Initially, for each of the variables, separately for control and experiment, the null hypothesis was tested, in which the results of the variable would be the same for the 3 groups defined by the evaluation times (2, 4 and 6 weeks) compared with the alternative hypothesis that the results would not all be the same. In case of rejection of the null hypothesis, the evaluations were compared two by two. Then, for each variable, in each of the evaluations (2, 4 and 6 weeks), the null hypothesis that the results of the control and experiment groups would be the same was tested, compared with the alternative hypothesis of different results.

## Results

During the surgical procedure and electric stimulation, 8 of the animals died, 2 from the control group and 6 from the experiment group. These deaths were related to the anesthetic procedure and metabolic recovery from the medications used. A value was removed from each experiment and control group due to the early interruption of the tensiometric test, thus not forming an adequate graph for measurement. A value was excluded from the experiment groups with 6 weeks of maximum tension and loading due to the slipping of the part that led to the formation of an inadequate graph for mechanical evaluation.

**Figure 3:**
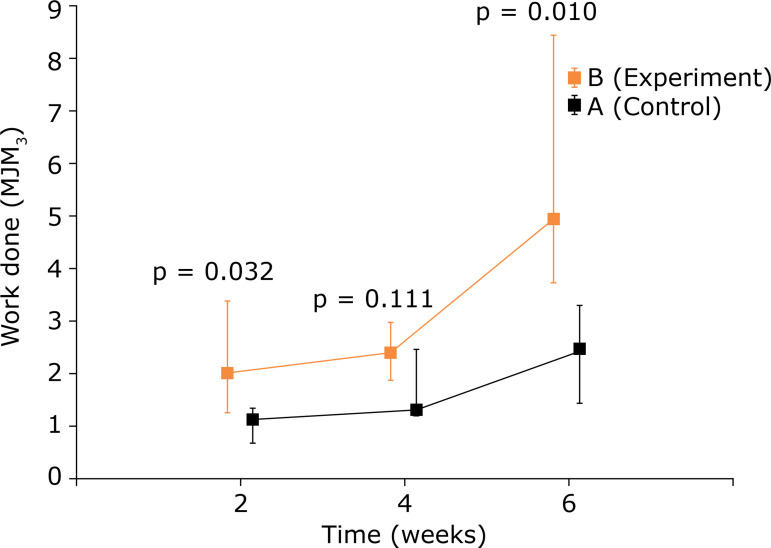
Comparison between control group and experiment group for work performed (MJ/m^3^) - used medians and maximum and minimum values.

In the analysis of the work, the intragroup observation showed improvement, with the passage of time in both groups, more important in the experiment group. There was a significant difference in the comparison between the groups in the second (p = 0.032) and sixth week (p = 0.010), with better results for the electrostimulated group. In the fourth week, although the group showed better performance, it was not significant (p = 0.111) (Table 1 and Fig. 3).

**Table 1:** Comparative analysis, intragroups, of work performed (MJ/m^3^).

Group	Evaluation	Work							p*			
		n	Average	Median	Minimum	Maximum	Standard deviation		2 × 4 × 6 weeks	2 × 4 weeks	2 × 6 weeks	4 × 6 weeks
A	2 weeks	5	1.05	1.12	0.65	1.28	0.24					
	4 weeks	5	1.63	1.28	1.17	2.41	0.56					
	6 weeks	6	2.48	2.44	1.43	3.28	0.65		0.006	0.135	< 0.001	0.03
B	2 weeks	4	2.15	1.98	1.26	3.37	0.88					
	4 weeks	4	2.39	2.36	1.87	2.97	0.48					
	6 weeks	4	5.52	4.92	3.72	8.52	2.08		0.023	0.543	0.003	0.007

* Kruskal–Wallis nonparametric test p < 0.05 ** A = Control group; B = Experiment group.

**Figure 4:**
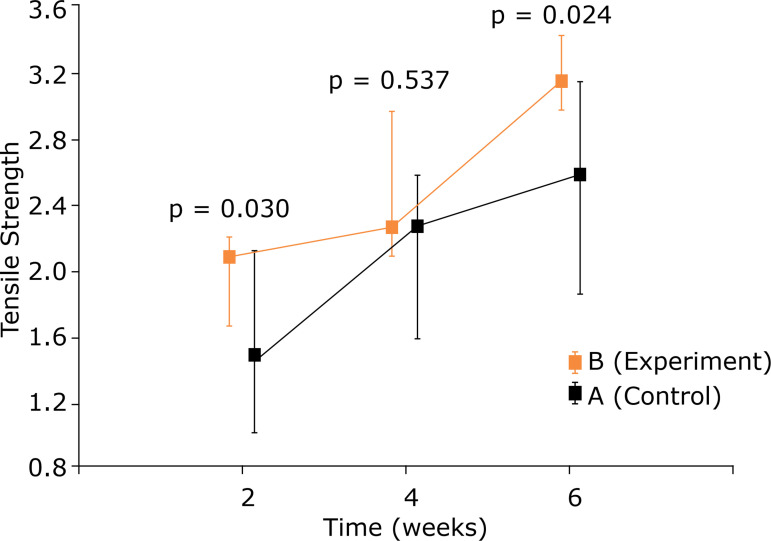
Comparison between control group and experiment group for maximum stress (MPa) - used medians and maximum and minimum values.

The maximum tension, compared intragroup, showed a gain at the times in question. The evaluation after 2 weeks showed a better result in the experiment group (p = 0.030). At 4 weeks, although this group still had the greatest gain, it was not significant, but became so again after 6 weeks (p = 0.024) (Table 2 and Fig. 4).

**Table 2:** Comparative analysis, intragroups, of maximum stress (MPa).

Group	Evaluation	Maximum Stress							p *			
		n	Average	Median	Minimum	Maximum	Standard deviation		2 × 4 × 6 weeks	2 × 4 weeks	2 × 6 weeks	4 × 6 weeks
A	2 weeks	6	1.49	1.46	1.00	2.10	0.36					
	4 weeks	6	2.18	2.27	1.56	2.56	0.39					
	6 weeks	7	2.50	2.55	1.83	3.13	0.45		0.004	0.004	< 0.001	0.174
B	2 weeks	5	1.94	2.06	1.64	2.18	0.27					
	4 weeks	5	2.41	2.24	2.07	2.94	0.37					
	6 weeks	4	3.16	3.13	2.96	3.40	0.19		0.006	0.018	< 0.001	0.005

* Kruskal–Wallis nonparametric test p < 0.05 ** A = Control group; B = Experiment group.

**Figure 5:**
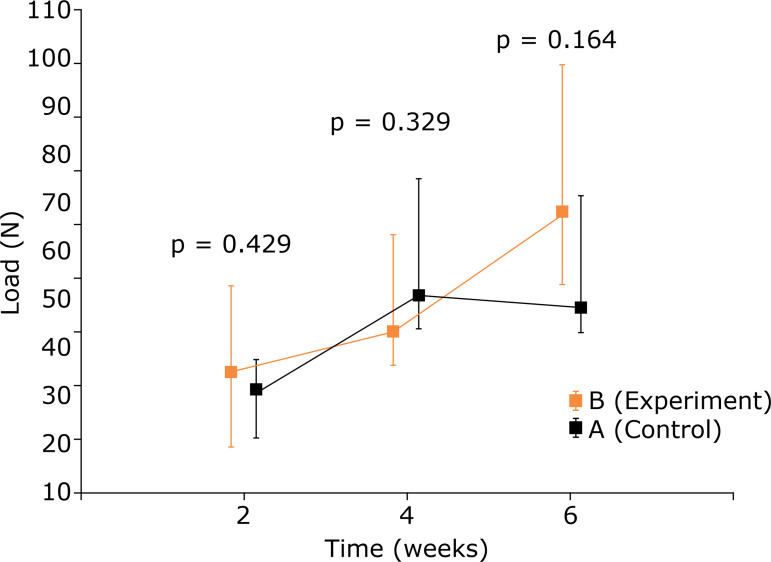
Comparison between control group and experiment group for maximum load (N) - used medians and maximum and minimum values.

**Figure 6:**
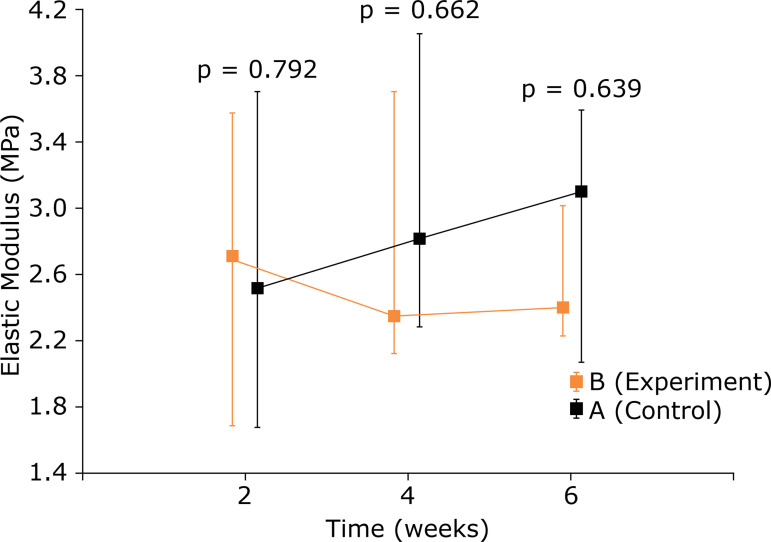
Comparison between control group and experiment group for elastic modulus (MPa) - used medians and maximum and minimum values.

An analysis of the maximum load in an intragroup comparison showed a significant improvement in over time in the experimental group. In the control group, there was gain until the fourth week. From the fourth to the sixth week there was no significant change (p = 0.641).The comparison between the control and experiment groups showed no significant difference at the three times in question (p > 0.05) (Table 3 and Fig. 5). The analysis of the elastic modulus showed the same findings (Table 4 and Fig. 6).

**Table 3:** Comparative analysis, intragroups, of maximum load (N).

Group	Evaluation	Maximum Load							p *			
		n	Average	Median	Minimum	Maximum	Standard deviation		2 × 4 × 6 weeks	2 × 4 weeks	2 × 6 weeks	4 × 6 weeks
A	2 weeks	6	30.0	31.1	21.6	37.7	6.2					
	4 weeks	6	55.0	51.5	44.0	74.9	11.2					
	6 weeks	7	54.5	48.5	43.1	70.9	11.9		0.003	< 0.001	< 0.001	0.641
B	2 weeks	5	36.3	35.2	19.8	53.1	12.2					
	4 weeks	5	48.9	43.9	36.7	63.9	11.8					
	6 weeks	4	72.0	68.0	53.3	98.7	20.0		0.018	0.045	0.001	0.059

* Kruskal–Wallis nonparametric test p<0.05 ** A = Control group; B = Experiment group.

**Table 4:** Comparative analysis, intragroups, of elastic modulus (MPa).

Group	Evaluation	Elastic Modulus						p* (2 × 4 × 6 weeks)
		n	Average	Median	Minimum	Maximum	Standard deviation	
A	2 weeks	6	2.56	2.52	1.66	3.71	0.73	
	4 weeks	6	2.95	2.82	2.27	4.05	0.76	
	6 weeks	7	2.91	3.11	2.05	3.59	0.71	0.558
B	2 weeks	5	2.70	2.72	1.68	3.58	0.74	
	4 weeks	5	2.67	2.35	2.13	3.69	0.62	
	6 weeks	5	2.53	2.40	2.23	3.01	0.31	0.932

* Kruskal–Wallis nonparametric test p < 0.05 ** A = Control group; B = Experiment group.

## Discussion

Rupture of the Achilles tendon is one of the most frequent tendon injuries of the lower limb. This tendon is important because of the pressure on the foot when walking, as it connects the calf muscles to the heel bone. Thus, recovery from this rupture is very important. The lesion has a bimodal age distribution with the first peak in patients between 25 and 40 years of age and the second peak in patients over 60 years of age. High energy injuries in sports are responsible for the first peak, while the second peak occurs in the elderly and is mainly associated with the spontaneous rupture of degenerative tendons^26^.

Since the Achilles tendon is the most important plantar flexor of the foot, its rupture can have a significant impact on its function. The treatment is intended to restore the tendon integrity and length so that patients can return to their activities with the intensity of strength that they enjoyed prior to the injury. Treatment can be either conservative with early functional rehabilitation or surgery. However, there is no consensus as to which is the best option^27^.

Controversy surrounds the best treatment for acute Achilles tendon ruptures. Multiple treatments present good results in the short and long term, with none being superior to the others if followed by a full early rehabilitation protocol to support the patient’s weight^28^.

Surgical complications are generally related to tendon or skin healing problems, caused mainly by poor local vascularization^1^,^2^,^4^,^5^,^7^.

Snyder *et al.* 9 conducted a review of the effect of electric stimulation in the healing of tissues and concluded that, despite their exact interference mechanisms in the healing of tissues, further studies were required. Although a wide variety of combinations of electric stimulation patterns hinder more conclusive studies, electric stimulation proved to be a safe and effective treatment for healing, improving the cutaneous perfusion of bones, tendons and ligaments.

Studies conducted *in vitro* with electric stimulation have shown a greater production of collagen and fibroblast migration^15^,^29^. This treatment increased the migration of mesenchymal stem cells in rats^30^. *In vitro* human fibroblasts with electric stimulation using a galvanic and high-voltage current showed a significant increase in the synthesis of proteins and DNA^17^.

A literature review suggested that transcutaneous electric stimulation improves wound healing, tendon repair and skin-flap viability. The authors argue that this is due to the increased production of substance P and the peptide genetically related to calcitonin, which increase local blood flow and improve healing^31^.

Salazar *et al*.^32^ studied rats and suggested that electroacupuncture could lead to better connectivity between the anterior hypothalamus and the amygdala, resulting in the better formation of mesenchymal stem cells in circulation. They also suggested a reduction in pain and an increase in serum IL-10. Since IL-10 is a cytokine that reduces inflammatory response, creating an environment favorable to the differentiation of regulating M2 macrophages and to regeneration and remodeling, this would explain the improved healing of Achilles tendons.

An experimental study in rats presented an evaluation of electric stimulation of 2, 15 and 120 Hz in the inflammatory process and expression of peripheral and central Cox2. It showed that electric stimulation, especially at 2 Hz, was effective in reducing edema in the inflammatory process, in addition to reducing hyperalgesia by regulating the expression of peripheral and central Cox2 receptors^33^.

Stimulation at 2 Hz and a voltage of 2–4 V without controlling the intensity in the Achilles tendons of rats led to greater synthesis of hydroxyproline and a better organization of the collagen fibers^34^. Thesefibers were examined under an electronic microscope and were thicker and more organized. This could account for the improved resistance^35^.

A high frequency could worsen the healing process. An experiment performed on the Achilles tendons of rats showed a reduction in the formation and alignment of collagen fibers with 100 Hz^10^. However, Rampazo *et al.* 36 used high-frequency (120 Hz) and high-voltage electric stimulation, comparing alternating currents (cathodic and anodic) and found no difference between groups in the formation and alignment of collagen and angiogenesis.

Several experimental works, not with tendons, but conducted to study electric stimulation in the healing of cutaneous wounds, showed greater production and migration of epithelial cells and greater collagen synthesis^14^, greater ATP synthesis and incorporation of amino acids in the skin proteins^16^. They described how electric stimulation is capable of activating the innate and adaptive immune system, improving the inflammatory response^37^.

It is difficult to understand and compare articles that present studies in which electric stimulation was applied. The application of 50 Hz, but without reporting the type of current used, revealed a greater number of cells, growth factors and greater maximum tension for rupture of the Achilles tendons of rats^38^. This same frequency ofnonpolarized current in the Achilles tendons of rats allowed an increase in angiogenesis and a greater number of fibroblasts with higher collagen density in the early stages of healing^39^.

Electric stimulation with a frequency of 10 Hz, albeit without explaining the details of the current, showed greater maximum tension in rat tendons. This study did not take into account the cross section of the tendon^40^. Nevertheless, a study of rats electrically stimulated at a low frequency (10 Hz) and with a positive (anodic) polarized current confirmed the increased maximum tension of the tendons. Electric stimulation at 10 Hz, with a high-voltage polarized current, developed greater resistance inthe group submitted to the anodic current than the control group with the cathodic current^41^.

The use of anodic or cathodic currents is another contradictory element. A study of rabbits with a 10 Hz polarized current showed better healing and resistance with a cathodic current in the third week and an anodic current in the fifth and eighth week^12^.

It should be noted that the polarized current increases the risk of electrolysis and tissue damage. For this reason, in this study, a nonpolarized current was used as a protocol. The most frequently used parameterization for the treatment of musculoskeletal and neuropathic pain is low frequency (2–10 Hz), and nonpolarized currents, with the production of several neurotransmitters such as beta-endorphins, serotonin, and norepinephrine^32^,^42^.

The results of the present study showed that the work conducted, energy absorption in MJ/m^3^ for tendon failure, which represents the tendon’s ability to absorb energy until its rupture, was significantly greater in the group with electric stimulation at 2 and 6 weeks (p = 0.032 and 0.010, respectively). It was possible to see more work done every week comparing control and experiment. However, at 4 weeks, it was not possible to show a significant difference. It is likely that this was related to the small sample and the variability in maximum and minimum values.

Regarding the maximum tension, the tendon’s capacity to support a load for rupture in MPa, it was also significant at 2 and 6 weeks in favor of the experiment group (p = 0.030 and 0.024, respectively). At 4 weeks, it was possible to see higher values of maximum tension in the experiment group, and there was no statistical significance. These data may have been adversely affected by the loss of the sample.

The maximum load, maximum strength for rupture in N showed a significant improvement in the intragroup evaluation over time. However, in the analysis between control and experiment there was no significant difference. When analyzing the maximum load, the tendon thickness value was not taken into account. Therefore, these are not good data for comparing different tissues.

The elastic modulus, which evaluates the elasticity/rigidity or tissues, showed no difference between the two groups. This enables the assumption that despite the tendon’s greater resistance following electric stimulation, it maintained its elasticity.

Despite the loss of the sample, it was verified that low-frequency electric stimulation with a nonpolarized current improved the resistance of the tendons. This may be due to the positive data described for the healing process and which will be motive behind next studies.

## Conclusion

Electric stimulation with a nonpolarized low-frequency (2 Hz) current increases the resistance of the Achilles tendon of rats, following tenotomy and suture with 2 and 6 weeks of evolution.

## References

[1] Järvinen TAH, Kannus P, Maffulli N, Khan KM (2005). Achilles tendon disorders: etiology and epidemiology. Foot Ankle Clin N Am.

[2] Sharma P, Maffulli N (2005). Tendon injury and tendinopathy. J Bone Joint Surg Am.

[3] Chen TM, Rozen WM, Pan WR, Ashton MW, Richardson MD, Taylor GI (2009). The arterial anatomy of the Achilles tendon: anatomical study and clinical implications. Clin Anat.

[4] Willits K, Amendola A, Bryant D, Mohtadi NG, Giffin JR, Fowler P, Kean CO, Kirkley A. (2010). Operative versus nonoperative treatment of acute achilles tendon ruptures: a multicenter randomized trial using accelerated functional rehabilitation. J Bone Joint Surg Am.

[5] Bhandari M, Guyatt GH, Siddiqui F, Morrow F, Busse J, Leighton RK, Sprague S, Schemitsch EH. (2002). Treatment of acute Achilles tendon ruptures a systematic overview and metaanalysis. Clin Orthop Relat Res.

[6] Wong J, Barrass V, Maffulli N (2002). Quantitative review of operative and nonoperative management of Achilles tendon ruptures. Am J Sports Med.

[7] Khan RJ, Fick D, Keogh A, Crawford J, Brammar T (2005). Treatment of acute Achilles tendon ruptures. J Bone Joint Surg.

[8] Cramp AFL, Gilsenan C, Lowe AS, Walsh DM (2000). The effect of high- and low-frequency transcutaneous electrical nerve stimulation upon cutaneous blood flow and skin temperature in healthy subjects. Clin Physiol.

[9] Snyder MJ, Wilensky JA, Fortin JD (2002). Current applications of electrotherapeutics in collagen healing. Pain Physician.

[10] Folha RAC, Pinfildi CE, Liebano RE, Rampazo ÉP, Pereira RN, Ferreira LM (2015). Can transcutaneous electrical nerve stimulation improve achilles tendon healing in rats?. Braz J Phys Ther.

[11] Hubacher J, Niemtzow RC, Corradino MD, Dunn JCY, Ha DH (2016). Standards for reporting electroacupuncture parameters. Med Acupunct.

[12] Ahmed AF, Elgayed S, Ibrahim I. (2012). Polarity effect of microcurrent electrical stimulation on tendon healing: biomechanical and histopathological studies. J Adv Res.

[13] Friedenberg ZB, Roberts Jr, Didizian NH, Brighton CT (1971). Stimulation of fracture healing by direct current in the rabbit fibula. J Bone Joint Surg Am.

[14] Alvarez OM, Mertz PM, Smerbeck RV, Eaglstein EW. (1983). The healing of superficial skin wounds is stimulated by external electrical current. J Investig Dermatol.

[15] Nessler JP, Mass DP (1987). Direct-current electrical stimulation of tendon healing in vitro. Clinl Orthop Relat Res.

[16] Cheng N, Van Hoof, Bockx E, Hoogmartens MJ, Mulier JC, De Ducker, Sansen WM, De Loecker (1982). Generation, protein synthesis, and membrane transport in rat skin. Clin Orthop Relat Res.

[17] Bourguignon GJ, Bourguignon LYW (1987). Electric stimulation of protein and DNA synthesis in human fibroblasts. FASEB J..

[18] Gault WR, Gatens PF (1976). Use of low intensity direct current in management of ischemic skin ulcers. Phys Ther.

[19] Carley PJ, Wainapel SF (1985). Low intensity direct current for wound healing. Arch Phys Med Rehabil.

[20] Al-Majed AA, Brushart TM, Gordon T (2000). Electrical stimulation accelerates and increases expression of BDNF and trkB mRNA in regenerating rat femoral motoneurons. Eur J Neurosci.

[21] Brushart TM, Hoffman PN, Royall RM, Murinson BB, Witzel C, Gordon T. (2002). Electrical stimulation promotes motoneuron regeneration without increasing its speed or conditio. J Neurosci.

[22] Willand MP, Nguyen M-A, Borschel GH, Gordon T (2016). Electrical stimulation to promote peripheral nerve regeneration. Neurorehabil Neural Repair.

[23] Gordon T (2016). Electrical Stimulation to enhance axon regeneration after peripheral nerve injuries in animal models and humans. Neurotherapeutics.

[24] Chen YS (2011). Effects of electrical stimulation on peripheral nerve regeneration. Biomedicine.

[25] Buitrago S, Martin TE, Tetens-Woodring J, Belicha-Villanueva A, Wilding GE (2008). Safety and efficacy of various combinations of injectable anesthetics in BALB/c mice. J Am Assoc Lab Anim Sci.

[26] Park S-H, Lee HS, Young KW, Seo SG (2020). Treatment of acute Achilles tendon rupture. Clin Orthop Surg.

[27] Clanton T, Stake IK, Bartush K, Jamieson MD (2020). Minimally invasive Achilles repair techniques. Orthop Clin N Am.

[28] Manent A, Lopes L, Coromina H, Santamaría A, Domínguez A, Llorens N, Sales M, Videla S (2019). Acute Achilles tendon ruptures: efficacy of conservative and surgical (percutaneous, open) treatment-A randomized, controlled, clinical trial. J Foot Ankle Surg.

[29] Cheng X, Gurkan UA, Dehen CJ, Tate MP, Hillhouse HW, Simpson GJ, Akkus O (2008). An electrochemical fabrication process for the assembly of anisotropically oriented collagen bundles. Biomaterials.

[30] Wang X, Gao Y, Shi H, Liu N, Zhang W, Li H. (2016). Influence of the intensity and loading time of direct current electric field on the directional migration of rat bone marrow mesenchymal stem cells. Front Med.

[31] Machado AFP, Santana EF, Tacani PM, Liebano RE. (2012). The effects of transcutaneous electrical nerve stimulation on tissue repair: A literature review. Can J Plast Surg.

[32] Salazar TE, Richardson MR, Beli E, Ripsch MS, George J, Kim Y (2017). Electroacupuncture promotes central nervous system-dependent release of mesenchymal stem cells. Stem Cells.

[33] Lee J-H, Jang K-J, Lee Y-T, Choi Y-H, Choi B-T (2006). Electroacupuncture inhibits inflammatory edema and hyperalgesia through regulation of cyclooxygenase synthesis in both peripheral and central nociceptive sites. Am J Chin Med.

[34] De Almeida MS, De Aro AA, Guerra FDR, Vieira CP, De Campos Vidal B, Rosa Pimentel (2012). Electroacupuncture increases the concentration and organization of collagen in a tendon healing model in rats. Connect Tissue Res.

[35] De Almeida, De Freitas, Oliveira LP, Vieira CP, Da Ré, Dolder MAH, Pimentel ER (2015). Acupuncture increases the diameter and reorganisation of collagen fibrils during rat tendon healing. Acupunct Med.

[36] Rampazo ÉP, Liebano RE, Pinfildi CE, Folha RAC, Ferreira LM (2016). High voltage pulsed current in collagen realignment, synthesis, and angiogenesis after achilles tendon partial rupture. Braz J Phys Ther.

[37] Korelo RIG, Kryczyk M, Garcia C, Naliwaiko K, Fernandes LC (2016). Wound healing treatment by high frequency ultrasound, microcurrent, and combined therapy modifies the immune response in rats. Braz J Phys Ther.

[38] Inoue M, Nakajima M, Oi Y, Hojo T, Itoi M, Kitakoji H (2015). The effect of electroacupuncture on tendon repair in a rat Achilles tendon rupture model. Acupunct Med.

[39] Ng GYF (2011). Comparing therapeutic ultrasound with microamperage stimulation therapy for improving the strength of Achilles tendon repair. Connect Tissue Res.

[40] Chan HKF, Fung DTC, Ng GYF (2007). Effects of low-voltage microamperage stimulation on tendon healing in rats. J Orthop Sports Phys Ther.

[41] Owoeye I, Spielholz NI, Fetto J, Nelson AJ. (1987). Low-intensity pulsed galvanic current and the healing of tenotomized rat Achilles tendons: preliminary report using load-to-breaking measurements. Arch Phys Med Rehabil.

[42] Zhang R, Lao L, Ren K, Berman BM (2014). Mechanisms of acupuncture–electroacupuncture on persistent pain. Anesthesiology.

